# LIPUS‐SCs‐Exo promotes peripheral nerve regeneration in cavernous nerve crush injury‐induced ED rats via PI3K/Akt/FoxO signaling pathway

**DOI:** 10.1111/cns.14256

**Published:** 2023-05-08

**Authors:** Kun Ye, Zitaiyu Li, Yinghao Yin, Jun Zhou, Dongjie Li, Yu Gan, Dongyi Peng, Ming Xiao, Liangyu Zhao, Yingbo Dai, Yuxin Tang

**Affiliations:** ^1^ Department of Urology The Fifth Affiliated Hospital of Sun Yat‐Sen University Zhuhai Guangdong China; ^2^ Guangdong Provincial Key Laboratory of Biomedical Imaging The Fifth Affiliated Hospital, Sun Yat‐Sen University Zhuhai Guangdong China; ^3^ Department of Urology Xiangya Hospital, Central South University Changsha China; ^4^ Department of Urology The Third Xiangya Hospital of Central South University Changsha China

**Keywords:** erectile dysfunction, exosomes, low‐intensity pulsed ultrasound, nerve regeneration, Schwann cells

## Abstract

**Objective:**

Clinical treatment of erectile dysfunction (ED) caused by cavernous nerve (CN) injury during pelvic surgery is difficult. Low‐intensity pulsed ultrasound (LIPUS) can be a potential strategy for neurogenic ED (NED). However, whether Schwann cells (SCs) can respond to LIPUS stimulation signals is unclear. This study aims to elucidate the signal transmission between SCs paracrine exosome (Exo) and neurons stimulated by LIPUS, as well as to analyze the role and potential mechanisms of exosomes in CN repair after injury.

**Methods:**

The major pelvic ganglion (MPG) neurons and MPG/CN explants were stimulated with LIPUS of different energy intensities to explore the appropriate LIPUS energy intensity. The exosomes were isolated and purified from LIPUS‐stimulated SCs (LIPUS‐SCs‐Exo) and non‐stimulated SCs (SCs‐Exo). The effects of LIPUS‐SCs‐Exo on neurite outgrowth, erectile function, and cavernous penis histology were identified in bilateral cavernous nerve crush injury (BCNI)‐induced ED rats.

**Results:**

LIPUS‐SCs‐Exo group can enhance the axon elongation of MPG/CN and MPG neurons compared to SCs‐Exo group in vitro. Then, the LIPUS‐SCs‐Exo group showed a stronger ability to promote the injured CN regeneration and SCs proliferation compared to the SCs‐Exo group in vivo. Furthermore, the LIPUS‐SCs‐Exo group increased the Max intracavernous pressure (ICP)/mean arterial pressure (MAP), lumen to parenchyma and smooth muscle to collagen ratios compared to the SCs‐Exo group in vivo. Additionally, high‐throughput sequencing combined with bioinformatics analysis revealed the differential expression of 1689 miRNAs between the SCs‐Exo group and the LIPUS‐SCs‐Exo group. After LIPUS‐SCs‐Exo treatment, the phosphorylated levels of Phosphatidylinositol 3‐kinase (PI3K), protein kinase B (Akt) and forkhead box O (FoxO) in MPG neurons increased significantly compared to negative control (NC) and SCs‐Exo groups.

**Conclusion:**

Our study revealed that LIPUS stimulation could regulate the gene of MPG neurons by changing miRNAs derived from SCs‐Exo, then activating the PI3K‐Akt‐FoxO signal pathway to enhance nerve regeneration and restore erectile function. This study had important theoretical and practical significance for improving the NED treatment.

## Graphical abstract

Exosomes derived from SCs with LIPUS pretreatment can promote the outgrowth of the nerve axons. Exosomal miRNA sequencing and target cell neuronal mRNA sequencing suggested that PI3K‐Akt‐FoxO signaling pathway is a key regulator of nerve regeneration. This finding provides an unprecedented perspective for the translational medicine in the field of nerve injury ED.

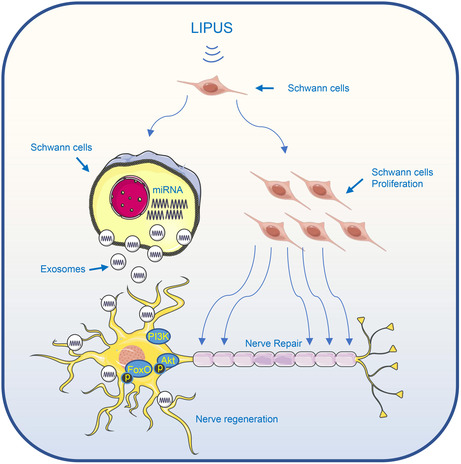

## 1 INTRODUCTION

Erectile dysfunction (ED) is a chronic clinical disease that seriously affects overall quality of life.^1^ ED may result from alterations in any component of the erectile response, including organic, relational, and psychological.^2^ The primary ED treatments include lifestyle modification, underlying primary disease treatment, phosphodiesterase type 5 inhibitors (PDE5‐Is), injected vasodilator agents, and penile prosthesis implantation.^2^, ^3^ PDE5‐Is is the first‐line treatment regimen for ED, and despite its remarkable efficacy, neurogenic ED due to trauma or surgery is often refractory to these drugs.^4^ Neurogenic erectile dysfunction (NED) is caused by central, peripheral, or both neurologic compromise or dysfunction.^5^, ^6^ Pelvic surgeries, such as radical prostatectomy (RP), are prone to damage the cavernous nerve (CN), leading to NED due to Wallerian degeneration and subsequent corpora cavernosa denervation following CN injury.^7^, ^8^ A new strategy to prevent the deterioration of erectile function secondary to nerve damage is urgently needed for this patient population.

Recently, clinical practice has gradually attempted to employ exogenous mechanical stimulation and microenergy to treat ED.^9^, ^10^, ^11^ Low‐intensity pulsed ultrasound (LIPUS) has an extremely small thermal effect and is frequently employed in rehabilitation medicine.^12^ In a multicenter clinical study, Cui et al. demonstrated that LIPUS could safely and effectively treat patients with mild to moderate ED.^13^ Chiang and Peng et al. speculated that LIPUS might target Schwann cells (SCs) to promote CN regeneration, thereby treating NED.^14^, ^15^

SCs are myelinating glial cells of the peripheral nervous system (PNS) and are a key feature of regeneration after peripheral nerve injury (PNI).^16^ Following PNI, SCs are released from degenerated nerves, rapidly activated by nerve injury‐induced signals, and then actively participate in axon regeneration.^17^ Dedifferentiated SCs can repair damaged nerves by recruiting macrophages and secreting neurotrophic factors during repair.^18^ A recent study revealed that LIPUS could up‐regulate SC pro‐myelination indicators.^19^ An additional study discovered that LIPUS could repair damaged nerves by promoting SCs proliferation.^20^ However, how SCs sense the effects of mechanical forces and the subsequent effects on neural regeneration remains unclear.

Neurons in the nervous system maintain their structural and functional integrity through communication with other neurons and glial cells.^21^ Exosomes (Exo) play a crucial role in intercellular communication. Exosomes are extracellular vesicles with a diameter of 30–150 nm secreted by cells; their main components are phospholipid bilayers, and they carry various biomolecules, including proteins, lipids, and RNA.^22^ When exosomes are internalized by other cells, their cargoes are transferred, affecting the recipient cell's phenotype. Therefore, exosomes are necessary mediators of intercellular communication.^22^ Several studies have demonstrated that SCs‐derived exosomes can promote nerve regeneration by transferring microRNAs (miRNAs).^23^, ^24^, ^25^ Furthermore, the exogenous mechanical force can enhance this effect,^26^ but the exact mechanism remains unclear.

This study aims to extract primary SCs from CN and examine the effects of SC‐derived exosomes (with or without LIPUS pretreated) on axonal regeneration and erectile function in vivo and in vitro. Furthermore, we intend to use high‐throughput sequencing and bioinformatics to identify and demonstrate the key signaling pathways in that LIPUS‐SCs‐Exo affects nerve regeneration. These results may open new avenues for exploring therapeutic approaches for NED.

## 2 MATERIALS AND METHODS

### 2.1 Animals

In this study, male Sprague Dawley (SD) rats were purchased from Guangdong Provincial Medical Laboratory Animal Center. Animal experiments were performed following the Guide for the Care and Use of Laboratory Animals, a publication of the National Institutes of Health. Animals were housed under pathogen‐free conditions in the Guangdong Key Laboratory of Biomedical Imaging.

### 2.2 Primary Schwann Cell Culture and purification

SC primary cultures were obtained from the CN of male SD rats (10–12 weeks old, 300–350 g). The CN was dissociated and dissected using microscopic instruments. The vessels and connective tissue attached to the CN were carefully dissected in pre‐chilled sterile Hank's balanced salt solution. The dissected CN was separated into small pieces and digested with a solution containing 0.1% collagenase type 1 (Gibco) and 0.25% trypsin (ThermoFisher, USA), then shaken at 37°C for 30 min to obtain a single‐cell suspension. After centrifugation, the single‐cell suspension was filtered using a 100 μm pore size cell strainer to obtain a cell pellet. Then, the cell pellet was resuspended in high glucose (4.5 g/L) Dulbecco's Modified Eagle Medium (DMEM) (Gibco) containing 10% exosome‐free fetal bovine serum (FBS) (Gibco). The cell suspension was placed in a culture dish treated with poly‐L‐lysine (Sigma) and laminin (Sigma) solution and cultured in a cell incubator. After culturing for 24 h, the old medium was replaced with DMEM containing FBS and cytosine arabinoside (Ara‐C) (Sigma) to remove fibroblasts, then cultured in a cell incubator. After culturing for 48 h, the old medium was replaced with DMEM containing 10% FBS, 1% penicillin/streptomycin (Gibco), 2 μM forskolin (Sigma), and 10 ng/mL human heregulin β1(PeproTech). Numerous high‐purity SCs can be obtained by expanding and culturing in an incubator. Double immunofluorescence staining of SC cultures based on SC markers S100β (1:100, ab52642, Abcam) and p75^NTR^ (1:100, ab245134, Abcam). Nuclei were stained with 4′,6‐diamidino‐2‐phenylindole (DAPI) solution (Sigma). Primary SC culture purity was measured by counting the number of S100β and p75^NTR^ double‐positive cells.

### 2.3 The culture and neurite growth measurement of major pelvic ganglion (MPG) neurons and MPG/CN explant

**FIGURE 1:**
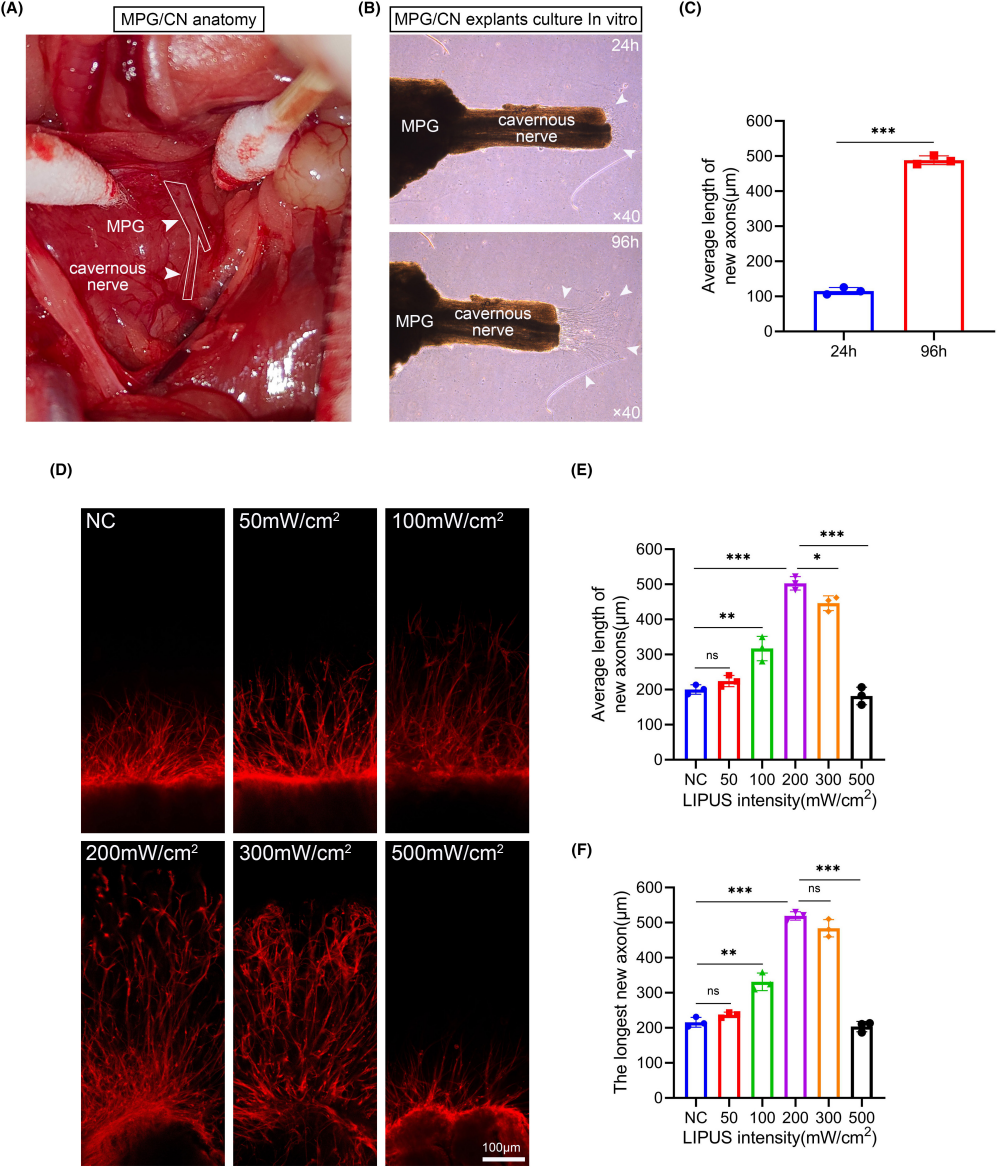
The effect of LIPUS at different energy intensities on the axonal growth of MPG/CN explants (A) Representative images of MPG and CN anatomy. (B) New neurites grew at the CN terminals as the culture time increased. Quantification of the average length of new axons is shown in panel (C). Data presented as mean ± SD with three replicates. ***p < 0.001.(D) Representative images of axonal growth length of MPG/CN explants stimulated by LIPUS with different energy intensities. New axons were stained for β3‐tubulin(red). (Scale bar = 100 μm.) Quantification of the average length and the longest length of new axons are shown in panel (E) and panel (F). Data presented as mean ± SD with three replicates. Ns, no significant difference; *p < 0.05; **p < 0.01; ***p < 0.001. CN, cavernous nerve; LIPUS, low‐intensity pulsed ultrasound; MPG, major pelvic ganglion; NC, negative control.

MPG/CN explants were harvested as previously described.^27^ Briefly, the bilateral MPG/CN complexes were isolated and excised. The anatomy of MPG/CN complexes is shown in Figure 1A. MPG/CN complexes were placed in a six‐well plate and then covered with 50 μL 10 mg/mL Matrigel (Corning). After incubation at 37°C for 5 min, 3 mL of complete DMEM was added to each group. MPG/CN explants were fixed in 4% paraformaldehyde (PFA) after three days of culture. Immunofluorescence detection was performed using β3‐tubulin (1:200, #5568, CST) antibody. The average and longest lengths of new axons extending from the end of the CN were measured to evaluate the axon elongation.

The dissected MPG was cut into small pieces, digested with a solution containing 0.3% collagenase type 1 and 0.25% trypsin, and then shaken at 37°C for 30 min to obtain a single‐cell suspension. The single‐cell suspension was filtered using a 100 μm pore size cell strainer and centrifuged to obtain the cell pellets. Then, cell pellets were resuspended in high glucose (4.5 g/L) DMEM containing 10% exosome‐free FBS. The cell suspension was placed in a culture dish treated with poly‐L‐lysine and laminin solution and cultured in a cell incubator at 37°C and 5% CO_2_. MPG neurons were cultured alone or with SCs (with or without LIPUS treatment) for 72 h to evaluate the SC effect on axon growth. Additionally, MPG neurons were pretreated with 10 μM LY294002 (MCE) or 1 μM MK2206 (SelleckChem) before LIPUS‐SCs‐Exo treatment for mechanism verification research to evaluate exosome effect on axon growth. Immunofluorescence detection was performed using β3‐tubulin (1:800, #5568, CST) antibody. The longest and total neurite lengths of neuronal body edge were measured to evaluate the neurite elongation.

### 2.4 LIPUS treatment

SCs and MPG/CN explants were treated using the LIPUS system (SXUltrasonic, Shenzhen, China). The MPG/CN explants were divided into six groups and treated with LIPUS at different intensities (0, 50, 100, 200, 300, and 500 mW/cm^2^; pulsed frequency: 1.0 MHz; duty cycle: 20%; and 10 min/d) for three times (2, 24, and 48 h) after seeding. SCs were treated with LIPUS (80 mW/cm^2^, 1 MHz, and 10 min/d) three times (2, 24, and 48 h) after seeding for cell biological function assay. Exosomes were isolated from cell supernatants after 72 h.

### 2.5 Cell viability assay and cell proliferation assay

The 3‐(4,5‐dimethylthiazol‐2‐yl)‐2,5‐diphenyltetrazolium bromide (MTT) assay was used to determine the effect of LIPUS at different energy intensities on SCs viability. Briefly, 2 × 10^4^ cells/well were inoculated on a 96‐well plate. The SCs were treated with LIPUS of different energy intensities (0, 20, 40, 60, 80, 100, and 200 mW/cm^2^; 10 min/d) three times. The cells were mixed with 20 μL MTT solution (5 mg/mL, Sigma) and incubated at 37°C for 4 h. Then, dimethyl sulfoxide was added to each pore. The absorbance value was measured at 570 nm (A570) using the microplate reader.

The cell proliferation was assessed using the Cell‐Light™ EdU Kit (Ribobio, Guangzhou, China). After 48 h of LIPUS treatment, the cells were incubated in complete DMEM containing 50 μM 5‐ethynyl‐2′‐deoxyuridine (EdU) for 24 h. SCs were labeled with Apollo and Hoechst staining. The cell proliferation was analyzed using a fluorescence microscope (Olympus).

### 2.6 Exosome purification and characterization

Exosomes were isolated and purified from LIPUS‐stimulated SCs (LIPUS‐SCs‐Exo) and non‐stimulated SCs (SCs‐Exo). Briefly, 1.5 × 10^7^ SCs (or LIPUS‐SCs) were cultured in T75 flasks with the exosome‐free medium. After 48 h of cell culture, the medium was collected for continuous centrifugation (300× *g* for 10 min, 2000× *g* for 10 min, and 10,000× *g* for 30 min at 4°C). The supernatant was filtered through a 0.22 μm filter and ultracentrifuged at 100,000× *g* for 70 min at 4°C. The exosome‐containing pellet was washed in phosphate buffer saline (PBS) and then ultracentrifuged at 100,000 *g* for 70 min at 4°C to collect exosomes. Exosomes were characterized using Transmission Electron Microscope (TEM, HITACHI H‐7650), Western blot, and Nanoparticle Tracking Analysis (NTA, MALVERN). The exosomes’ concentration was measured using NTA and Bicinchoninic Acid (BCA) assays (Solarbio). The exosome markers (TSG101, Alix, HSP70, and CD63) were identified using a western blot.

### 2.7 Internalization assays

Purified exosomes were labeled with a PKH26 kit (Sigma) to assess exosome endocytosis by cells or tissues. Briefly, 250 μL of exosomes were diluted with PBS and mixed with 250 μL of Diluent C. Then, 2 μL of PKH26 dye was added to 500 μL of Diluent C. The final PKH26 solution was mixed with the exosome solution prepared above for 5 min. The excess dye was neutralized by adding 1 mL of 1% bovine serum albumin. PKH26‐labeled exosomes were obtained by ultracentrifugation and resuspended in PBS. The diluted PKH26 solution was mixed with PBS as a negative control. PKH26‐labeled exosomes were co‐cultured with MPG neurons in serum‐free medium in vitro. Moreover, PKH26‐labeled exosomes were injected into MPG/CN in vivo. The exosome internalized MPG neurons or CN tissues were immunofluorescence stained with phalloidin (Abclonal, China); nuclei were stained with DAPI nuclear and observed under a laser scanning confocal microscope (ZEISS).

### 2.8 ED model establishment

Adult male SD rats (12‐week‐old) were used in this study. The bilateral CN crush injury (BCNI) model was established as described previously.^28^ All rats were divided into the sham‐operation group (NC), BCNI group, BCNI+SCs‐Exo group, and BCNI+LIPUS‐SCs‐Exo group. Briefly, an incision of approximately 4 cm was made in the skin of the lower abdominal midline. Then, the bladder and prostate were fully exposed, and the CN was carefully isolated. A hemostatic forceps tip 5 mm distal to the MPG was applied for complete closure for 1 min. After two days, apomorphine (100 μg/kg, Macklin) was used to screen the BCNI‐induced ED rats with successful modeling. Three days later, equal volumes of 20 μL PBS, SCs‐Exo, and LIPUS‐SCs‐Exo were injected into the proximal CN injury site at multiple points. Based on the results of exosomes concentration dependent experimental, the concentration of exosomes was 1 × 10^8^ particles/mL. After four weeks, all rats were examined for ED, and the penis and CN were harvested for follow‐up studies.

### 2.9 Erectile function measurement

Cavernosal hemodynamic changes were evaluated as previously described.^28^ The rats were exposed to MPG and CN through a median abdominal incision after anesthesia. The intracavernous pressure (ICP) was measured using a 25 G needle inserted into the corpus cavernosum connected to sterile heparinized saline. However, another needle was inserted into the left carotid artery to monitor changes in mean arterial pressure (MAP). The CN was stimulated using bipolar hook electrodes with the following parameters: voltage at 1.0 V, frequency at 20 Hz, and pulse width at 5 ms. Approximately 1 min of stimulation was applied, with 5 min rest. The ICP and MAP were recorded and analyzed using the BL‐420 N system (TAIMENG). Erectile function was evaluated by comparing ICP to MAP ratio in each group.

### 2.10 Histological examination of corpus cavernosum of penis

Penile tissue was fixed with 4% paraformaldehyde, cut into 5 μm thick paraffin sections, and stained with hematoxylin and eosin (H&E) and Masson, according to kit instructions (Solarbio). The sections were dewaxed in xylene and rehydrated with various ethanol concentrations. In H&E staining, hematoxylin and eosin were used to stain the nucleus and cytoplasm. In Masson staining, ponceau red and aniline blue dyes were used to stain muscle and collagen tissues, respectively.

### 2.11 Immunofluorescence

The cell cultures and tissue slides were fixed with 4% paraformaldehyde, then permeabilized with Triton X‐100 (Solarbio) for 10 min, and blocked with 5% normal goat serum (Solarbio) for 30 min. The cell cultures and the tissue slides were incubated overnight at 4°C with primary antibodies, including β3‐tubulin, s100β, α‐SMA (1:200, ab7817, Abcam), CD31 (1:100, ab222783, Abcam), and eNOS (1:200, #32027, CST), and reacted with fluorescently conjugated secondary antibodies. The images were analyzed using a fluorescence microscope or laser scanning confocal microscope.

### 2.12 RNA sequencing and bioinformatics analysis

Total RNA was extracted from SCs‐Exo and LIPUS‐SCs‐Exo, and miRNA sequencing was conducted. The sequence and library preparation were performed by BGI Genomics Co., Ltd. RNA sequencing was conducted using the Illumina MGIseq2000 platform. Total RNA was extracted and isolated from exosomes, and only small RNAs of 18–30 nt were selected for library construction. A computational target prediction algorithm, miRWalk (http://mirwalk.umm.uni‐heidelberg.de/), was used to identify miRNA binding sites to predict the potential target genes of differential miRNAs. However, total RNA extracted from MPG neurons treated with SCs‐Exo and LIPUS‐SCs‐Exo was used for mRNA sequencing. The miRNA targets and mRNA pathways in the Kyoto Encyclopedia of Genes and Genomes (KEGG) were annotated. The predicted exosomal miRNA target and the target gene of neurons were intersected and analyzed using a VENN diagram. Additionally, principal component analysis (PCA), volcano, and heatmap were generated using R‐Studio.

### 2.13 Quantitative real‐time polymerase chain reaction (qRT‐PCR)

The qRT‐PCR was employed to verify the miRNA sequencing results by selecting miRNAs with log_2_ (Fold change) absolute value ≥3 and the top five miRNAs expressed in the exosomes (including 5 up‐regulated miRNAs and 5 down‐regulated miRNAs, respectively). Briefly, miRNAs were extracted from SCs‐Exo and LIPUS‐SCs‐Exo using a miRNA kit (Omega Biotek, China). The miRNA expression was measured using miRNA Universal SYBR qPCR Master Mix (Vazyme). The miRNA primers (one stem‐loop primer and one pair of qPCR primers) were designed by IGE Biotechnology Ltd (Guangzhou). The small nuclear RNA RNU6B (U6) was used as an internal control. The relative standard curve method (2^−∆∆Ct^) was utilized to determine the relative expression level. The primer sequences are shown in Table 1.

**TABLE 1:** Primer sequences.

miRNA	Sequence
rno‐let‐7c‐5p	F 5′‐GCGCGTGAGGTAGTAGGTTGT‐3′
	R 5′‐AGTGCAGGGTCCGAGGTATT‐3
rno‐miR‐34a‐5p	F 5′‐CGCGTGGCAGTGTCTTAGCT‐3′
	R 5′‐AGTGCAGGGTCCGAGGTATT‐3
rno‐miR‐23b‐3p	F 5′‐GCGATCACATTGCCAGGG‐3′
	R 5′‐AGTGCAGGGTCCGAGGTATT‐3′
rno‐miR‐375‐3p	F 5′‐GCGTTTGTTCGTTCGGCTC‐3′
	R 5′‐AGTGCAGGGTCCGAGGTATT‐3′
rno‐miR‐204‐5p	F 5′‐CGCGTTCCCTTTGTCATCCT‐3′
	R 5′‐AGTGCAGGGTCCGAGGTATT‐3′
rno‐miR‐423–3p	F 5′‐GAGCTCGGTCTGAGGCCC‐3′
	R 5′‐AGTGCAGGGTCCGAGGTATT‐3′
rno‐miR‐92b‐3p	F 5′‐GCGTATTGCACTCGTCCCG‐3′
	R 5′‐AGTGCAGGGTCCGAGGTATT‐3′
rno‐miR‐151‐5p	F 5′‐CGCGTCGAGGAGCTCACAG‐3′
	R 5′‐AGTGCAGGGTCCGAGGTATT‐3′
rno‐miR‐30a‐3p	F 5′‐GCGCTTTCAGTCGGATGTT‐3′
	R 5′‐AGTGCAGGGTCCGAGGTATT‐3′
rno‐miR‐196a‐5p	F 5′‐CGCGCGTAGGTAGTTTCATGTT‐3′
	R 5′‐AGTGCAGGGTCCGAGGTATT‐3′

### 2.14 Western blot

Protein was extracted from an exosome using a Total Exosome Protein Isolation Kit (ThermoFisher). The cultured cells were lysed in Radio Immunoprecipitation Assay Lysis Buffer (Biosharp). The protein sample was separated using sodium dodecyl sulphate‐polyacrylamide gel electrophoresis (SDS‐PAGE, Bio‐Rad) and transferred to polyvinylidene fluoride (PVDF) membranes (Millipore). The following antibodies were used as primary antibodies: Alix (ab186429, Abcam), TSG101 (A1692; Abclonal), CD63 (A19023, Abclonal), HSP70 (A12948; Abclonal), phospho‐PI3K (AP0854, Abclonal), PI3K (A11526, Abclonal), phospho‐Akt (AP0637, Abclonal), Akt (A11016, Abclonal), phospho‐FoxO1 (AP0172, Abclonal), FoxO1 (A2934, Abclonal), phospho‐FoxO3a (AP0684, Abclonal), FoxO3a (A0102, Abclonal), and β‐actin (AC026, Abclonal). Secondary antibodies were horseradish peroxidase (HRP) goat anti‐rabbit IgG (AS014, Abclonal) and goat anti‐mouse antibodies IgG (AS003, Abclonal). Visualization and analysis were performed using the iBright1500 system (Thermo).

### 2.15 Statistical analysis

The experimental data were presented as mean ± standard deviation (SD). Statistical analysis was performed using GraphPad Prism 8 software (GraphPad). All experiments were repeated three times, and all data were the average of three independent experiments. All data were tested for normality through Shapiro–Wilk test. The statistical significance between the two groups was analyzed using Kruskal‐Wallis test (abnormal distribution) or Student's t‐test (normal distribution). One‐way analysis of variance (ANOVA) was performed for statistical significance among multiple groups.

## 3 RESULTS

### 3.1 LIPUS enhances axonal growth from MPG/CN explants in an energy‐dependent manner

To investigate whether LIPUS influences axonal growth for MPG/CN explants in vitro, we established an in vitro model of MPG/CN culture. We dissected MPG with 2 mm CN attached and cultured them in vitro (Figure 1A). New neurites grew at the CN terminals as the culture time increased (Figure 1B,C). We discovered that LIPUS significantly improved axon growth after 72 h of treatment (Figure 1D). The average length of the new axons in the control group was 200.4 ± 13.6 μm. The average length of new axons in the LIPUS‐treated groups (50, 100, 200, 300, and 500 mW/cm^2^) was 224.2 ± 16.1 μm, 317.0 ± 34.7 μm, 502.9 ± 19.2 μm, 446.3 ± 21.0 μm, and 182.0 ± 25.0 μm, respectively (Figure 1E). Furthermore, we calculated the longest new axon length. The longest length of the new axon was 215.9 ± 13.9 μm in the control group. The longest length of new axons in the LIPUS‐treated groups (50, 100, 200, 300, and 500 mW/cm^2^) was 236.9 ± 7.6 μm, 331.2 ± 25.0 μm, 519.2 ± 12.1 μm, 483.9 ± 24.8 μm, and 203.5 ± 14.7 μm, respectively (Figure 1F). These results demonstrated that LIPUS enhances axonal growth in an energy‐dependent manner.

### 3.2 LIPUS promotes SCs viability and proliferation

**FIGURE 2:**
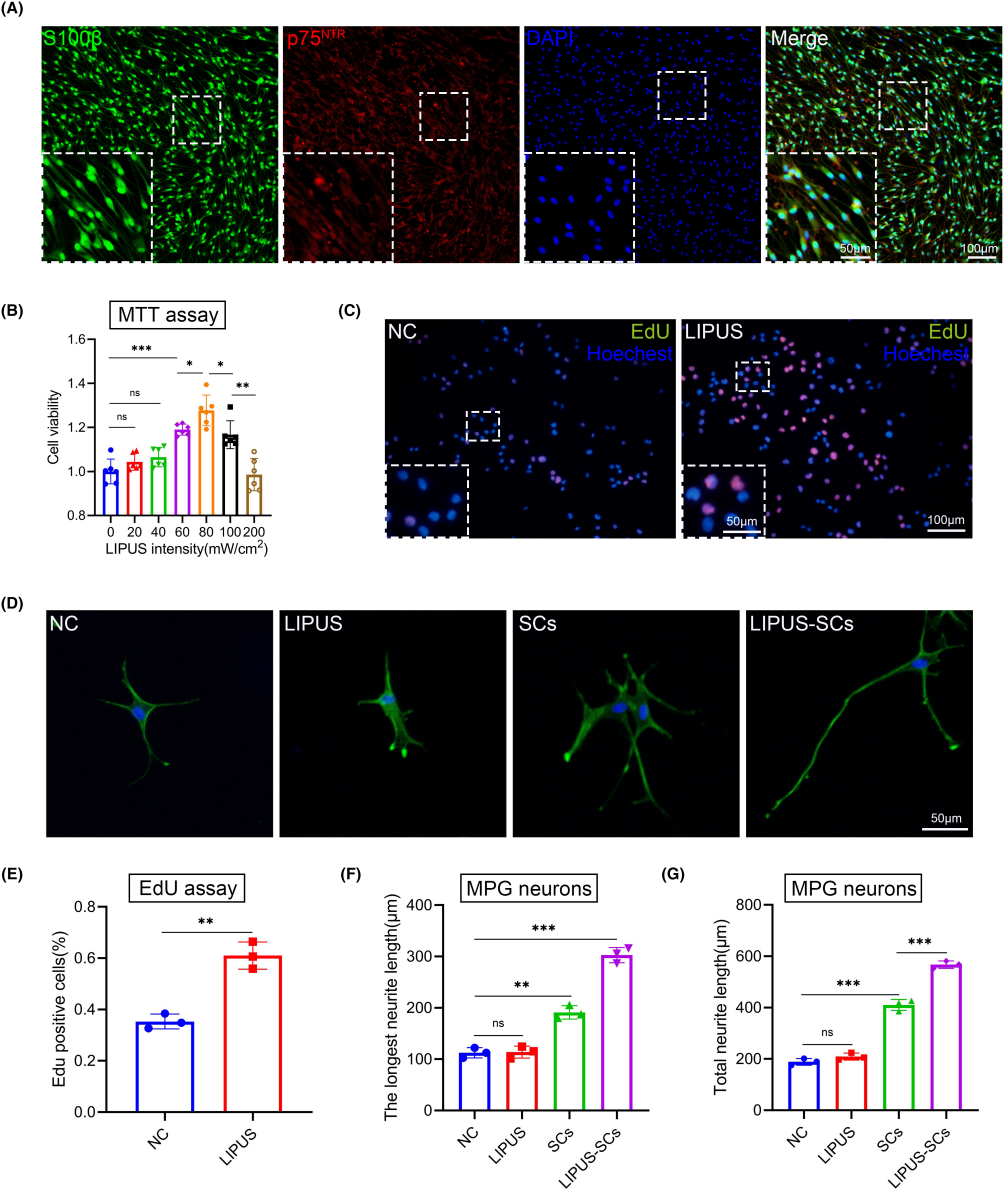
The effect of LIPUS on the SCs viability and proliferation. (A) Characterization of primary SCs from CN is shown by immunofluorescent staining for S100β (green), p75^NTR^ (red), DAPI (blue) and Merge file. (Scale bar = 100 μm.) (B) The viability of SCs stimulated by LIPUS with different energy intensities for 72 h was evaluated by MTT assay. The data were presented as mean ± SD. *n* = 3; Ns, no significant difference; *p < 0.05; ***p < 0.001. (C) SCs proliferation was assessed by EdU assay. (Scale bar = 100 μm.) (D) MPG neurons were assessed by immunofluorescent staining for β3‐tubulin (green) and DAPI (blue). (Scale bar = 50 μm.) Quantification of Edu positive cells is shown in panel (E). Data presented as mean ± SD with three replicates. **p < 0.01. Quantification of the longest neurite length and total neurite length of MPG neurons is shown in panel (F) and panel (G). Data presented as mean ± SD with three replicates. Ns, no significant difference; **p < 0.01; ***p < 0.001. CN, cavernous nerve; LIPUS, low‐intensity pulsed ultrasound; MPG, major pelvic ganglion; NC, negative control; SCs, Schwann cells.

To evaluate whether LIPUS affects cell viability and proliferation, we first isolated primary SCs from the CN of SD rats. The S100 and p75^NTR^ were identified as SCs using immunofluorescence staining (Figure 2A). Then, we treated SCs with different intensities of LIPUS (0, 20, 40, 60, 80, 100, and 200 mW/cm^2^) (Figure 2B). We discovered that LIPUS with appropriate energy intensity promoted the SCs viability more than the control group using the MTT assay. When the LIPUS energy intensity was set to 80 mW/cm^2^, the effect of promoting SCs viability peaked. Furthermore, we evaluated the effect of LIPUS (80 mW/cm^2^) on the SCs proliferation using an EdU assay (Figure 2C). The EdU percentage incorporation was 35.3 ± 2.9% in the control group but increased to 61.0 ± 5.3% in the LIPUS treatment group (*p* < 0.05) (Figure 2E). These findings indicated that LIPUS promotes SCs viability and proliferation.

### 3.3 LIPUS regulates MPG neuron neurite outgrowth

To explore the mechanisms by which LIPUS promotes MPG/CN explants neurite outgrowth, we divided the experiments into four groups: control group (NC), LIPUS group, SCs group, and LIPUS‐SCs group (Figure 2D). The untreated MPG neurons served as the control group. MPG neurons were treated with LIPUS (80 mW/cm^2^, 10 min/d) for three days in the LIPUS group. The SCs group means that SCs were co‐incubated with MPG neurons for three days. LIPUS‐pretreated SCs were co‐incubated with MPG neurons for three days in the LIPUS‐SCs group. The neurite length of MPG neurons did not differ significantly between the LIPUS (longest neurite length: 113.9 ± 11.6 μm; total neurite length: 209.1 ± 13.7 μm) and the control groups (longest neurite length: 112.4 ± 10.0 μm; total neurite length: 188.9 ± 13.0 μm). However, the neurite length of the SCs group (longest neurite length: 191.2 ± 13.0 μm; total neurite length: 410.3 ± 21.5 μm) was significantly increased than the control group. Moreover, the LIPUS‐SCs group (longest neurite length: 302.7 ± 14.9 μm; total neurite length: 567.5 ± 14.4 μm) had longer neurite lengths than the SCs group (p < 0.05) (Figure 2F,G). These findings indicated that SCs were the key factor for LIPUS to promote MPG neuron neurite outgrowth.

### 3.4 Characterization and internalization of exosomes

**FIGURE 3:**
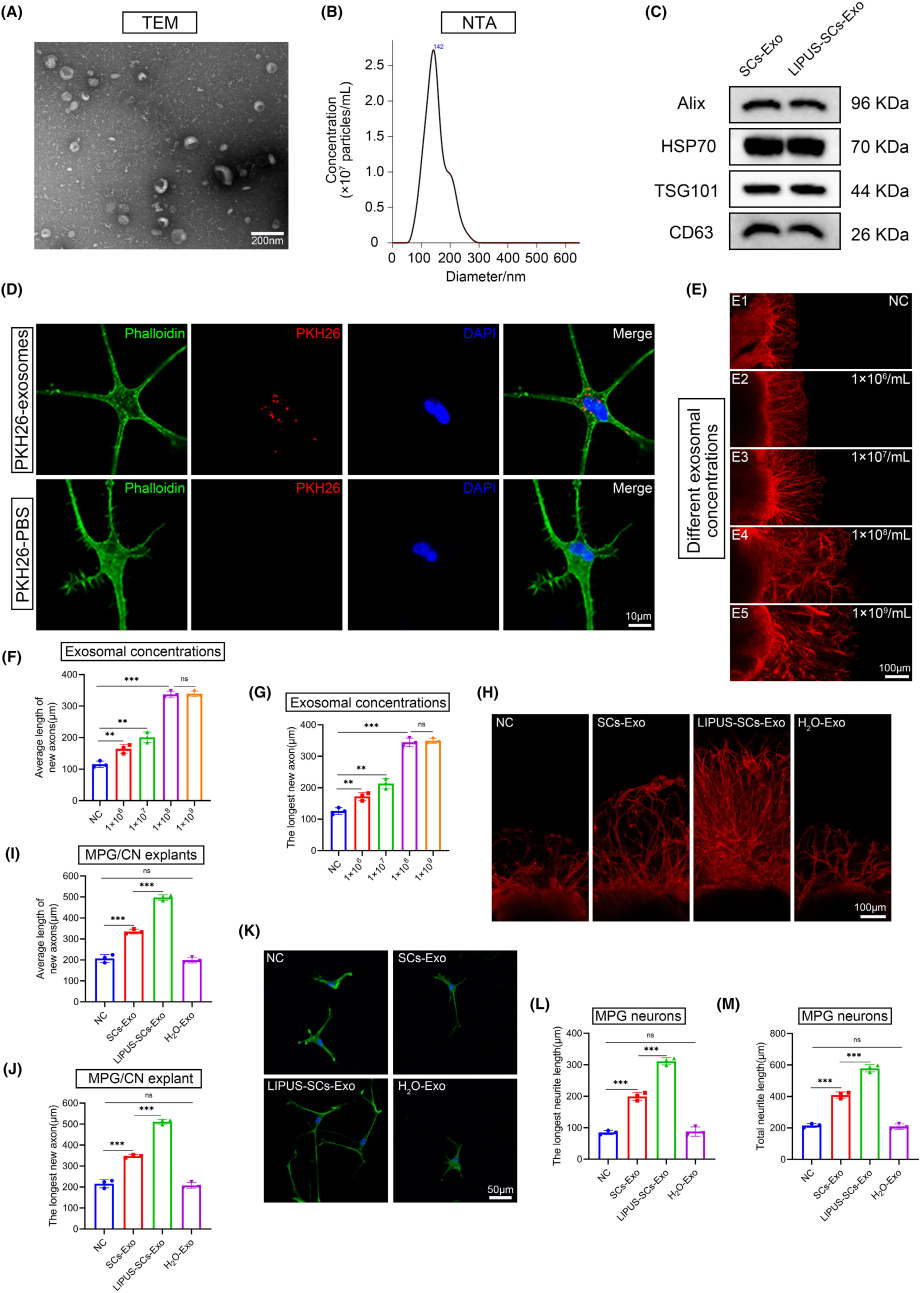
The effect of LIPUS‐SCs‐Exo on the neurite outgrowth in vitro. (A) TEM analysis of isolated exosomes. (Scale bar = 200 nm.) (B) The size of exosomes was detected by NTA. (C) Western blot analysis showing exosome markers Alix, HSP70, TSG101 and CD63. Panel (D) showed that PKH26(red)‐labeled exosomes were detected in cytoplasm and neurite of MPG neurons, and not in the negative control group. MPG neurons were immunofluorescent stained cytoskeletal staining by phalloidin (green) and DAPI (blue). (Scale bar = 10 μm.) (E) The effect of different concentrations of LIPUS‐SCs‐Exo(E1‐E5) on axonal growth in MPG/CN explants. Quantification of the average length and the longest length of new axons is shown in panel (F) and panel (G). Data presented as mean ± SD with three replicates. Ns, no significant difference; **p < 0.01; ***p < 0.001. Axonal elongation of MPG/CN explants(H) and MPG neurons(K) after 3 days in PBS, SCs‐Exo, LIPUS‐SCs‐Exo, LIPUS‐SCs‐Exo pre‐treated with H_2_O. Quantification of neurites is shown in panel (I), panel (J), panel (L) and panel (M). Data presented as mean ± SD with three replicates. Ns, no significant difference; **p < 0.01; ***p < 0.001. CN, cavernous nerve; Exo, exosomes; LIPUS, low‐intensity pulsed ultrasound; MPG, major pelvic ganglion; NC, negative control; NTA, Nanoparticle Tracking Analysis; SCs, Schwann cells; TEM, Transmission Electron Microscope.

**FIGURE 4:**
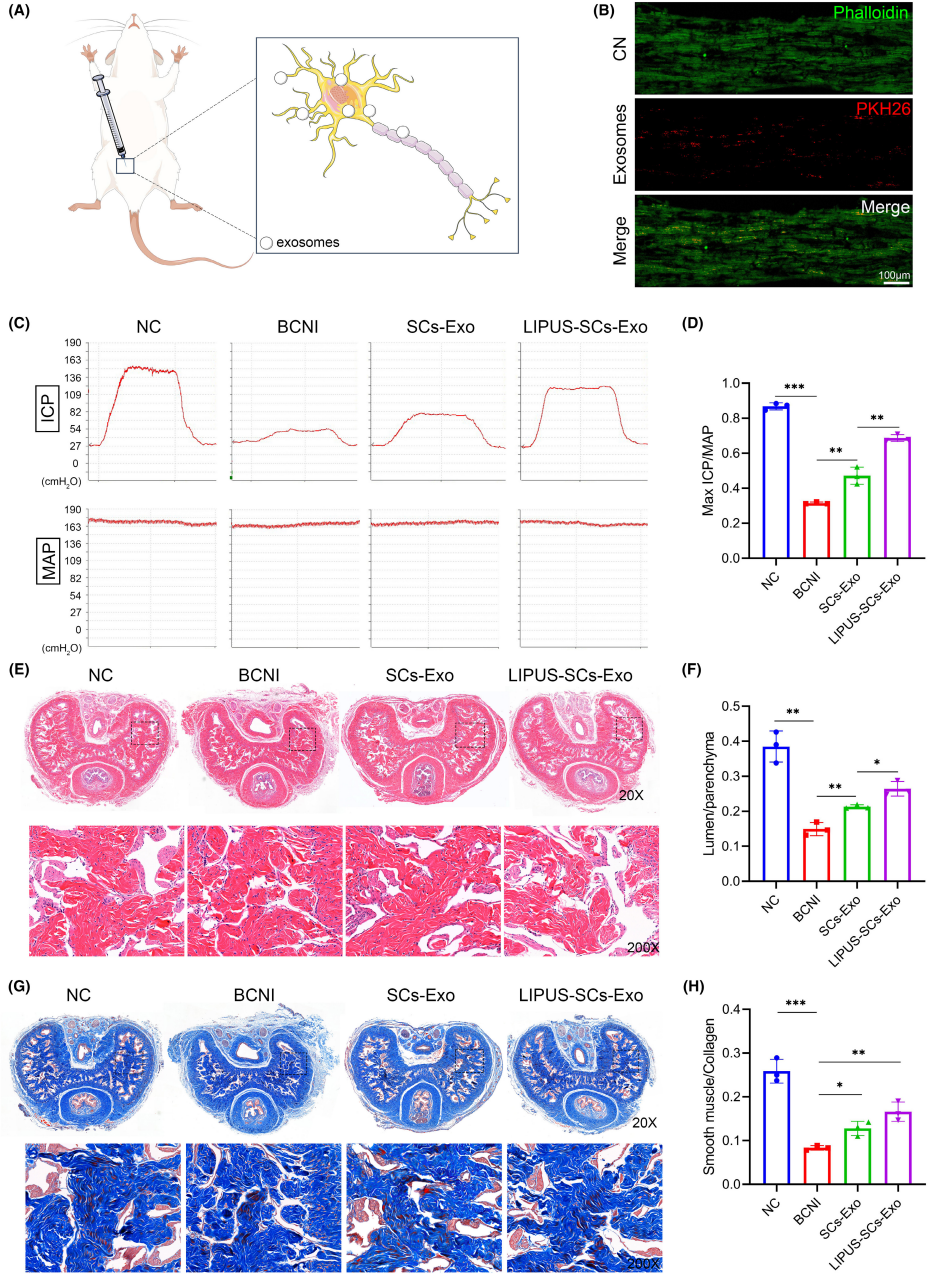
The effect of LIPUS‐SCs‐Exo on erectile function and histological alteration in vivo. (A) Schematic diagram of exosomes treatment. Panel (B) showed that PKH26(red)‐labeled LIPUS‐SCs‐Exo was detected in CN. CN were immunofluorescent stained cytoskeletal staining by phalloidin (green). (Scale bar = 100 μm.) (C) Representative images of hemodynamic changes after CN stimulation. (D) The maximum ICP/MAP ratio of each group was measured to evaluate erectile function. The data were presented as mean ± SD. *n* = 3; **p < 0.01; ***p < 0.001. (E) The morphological characteristics of corpus cavernosum were observed by H&E staining. (F) Semi‐quantitative analysis of the ratio of cavernous lumen to parenchyma. The data were presented as mean ± SD. *n* = 3; *p < 0.05; **p < 0.01. (G) Corpus cavernosum was evaluated by Masson's trichrome staining, smooth muscle (red), collagen (blue). (H) Semi‐quantitative analysis of the ratio of smooth muscle to collagen. The data were presented as mean ± SD. *n* = 3; *p < 0.05; **p < 0.01; ***p < 0.001. CN, cavernous nerve; Exo, exosomes; H&E, hematoxylin and eosin; ICP, intracavernous pressure; LIPUS, low‐intensity pulsed ultrasound; MAP, mean arterial pressure; SCs, Schwann cells.

Based on the above results, SCs may not only repair damaged nerves through self‐proliferation but also affect nerve regeneration through paracrine secretion. Lopez et al found that SCs‐derived exosomes promote axon growth in the PNS.^23^ To investigate how LIPUS‐SCs promote MPG neuron neurite outgrowth, we extracted exosomes derived from SCs (with or without LIPUS treatment). We observed the morphology of cup‐shaped exosomes using TEM (Figure 3A). NTA analysis demonstrated that the diameter of LIPUS‐SCs‐Exo peaked at 141.7 ± 1.1 nm (Figure 3B). Western blot results revealed that SCs‐Exo and LIPUS‐SCs‐Exo expressed exosomal markers Alix, TSG101, CD63, and HSP70(Figure 3C). After labeling LIPUS‐SCs‐Exo with PKH26, we co‐incubated LIPUS‐SCs‐Exo with MPG neurons to investigate whether MPG neurons can internalize exosomes. PKH26‐labeled LIPUS‐SCs‐Exo (a positive rate of 97.4 ± 1.2%) was detected in cytoplasm and neurite of MPG neurons after 24 h of immunofluorescence but not in the negative control group (a positive rate of 0%) (Figures 3D). Furthermore, we injected LIPUS‐SCs‐Exo into the MPG/CN of SD rats (Figure 4A), and 24 h later, immunofluorescence detected PKH26‐labeled LIPUS‐SCs‐Exo in the CN (Figure 4B). These findings suggested that MPG neurons and CN could internalize LIPUS‐SCs‐Exo in vivo and in vitro.

### 3.5 Exosomes derived from LIPUS‐SCs enhance neurite outgrowth in vitro

To identify the effectiveness of LIPUS‐SCs‐Exo on nerve regeneration, we examined its effect on MPG/CN explants and MPG neurons. First, we examined the effects of different LIPUS‐SCs‐Exo concentrations on axonal growth in MPG/CN explants (Figure 3E). We discovered that LIPUS‐SCs‐Exo promoted axonal growth in MPG/CN explants in a concentration‐dependent manner. When the exosome concentration reached 1 × 10^8^ particles/mL, the beneficial effect of promoting MPG/CN explant axonal growth was achieved (Figure 3F,G). Therefore, we set the exosome concentration to 1 × 10^8^ particles/mL for follow‐up studies. The SCs‐Exo group (average new axon length: 335.1 ± 11.0 μm; longest new axon: 347.2 ± 7.4 μm) significantly promoted axonal growth in MPG/CN explants than the control group (average new axon length: 207.1 ± 19.1 μm; longest new axon: 216.2 ± 19.4 μm) after 3 days. Additionally, the LIPUS‐SCs‐Exo group (average new axon length: 497.5 ± 13.2 μm; longest new axon: 511.1 ± 11.8 μm) had a beneficial effect on promoting the axonal growth of MPG/CN explants than the SCs‐Exo group. In contrast, when LIPUS‐SCs‐Exo was pretreated with H_2_O (H_2_O‐Exo), its effect on promoting neurite outgrowth was reversed (Figure 3H–J). These results indicated that only intact exosomes have the effect of promoting axon regeneration.

We evaluated further the effect of LIPUS‐SCs‐Exo on the axonal length of MPG neurons. Similar to the results of MPG/CN explants, the neurite length of MPG neurons in the LIPUS‐SCs‐Exo (longest neurite length: 311.0 ± 11.1 μm; total neurite length: 579.1 ± 22.8 μm) group was increased than that in the negative control group (longest neurite length: 85.1 ± 6.3 μm; total neurite length: 215.8 ± 12.3 μm) and SCs‐Exo group (longest neurite length: 199.6 ± 12.5 μm; total neurite length: 409.4 ± 19.6 μm). The H_2_O‐Exo group could counteract the axonal growth‐promoting effect of LIPUS‐SCs‐Exo (Figure 3K–M). These data indicated that SCs‐Exo can promote axonal regeneration, and LIPUS‐SCs‐Exo had a stronger effect.

### 3.6 LIPUS‐SCs‐Exo improves erectile function effectively in vivo

To evaluate whether LIPUS‐SCs‐Exo had a therapeutic effect on erectile function in BCNI rats, we treated BCNI rats with local injection of MPG/CN using SCs‐Exo and LIPUS‐SCs‐Exo (Figure 4A). We first measured ICP and MAP in rats after four weeks (Figure 4C). The Max ICP/MAP ratio was decreased in the BCNI group (0.31 ± 0.006) than in the control group (0.87 ± 0.020). The Max ICP/MAP was sensibly increased in both exosome treatment groups compared to the BCNI group. The LIPUS‐SCs‐Exo group (0.69 ± 0.020) had a beneficial treatment effect compared to the SCs‐Exo group (0.47 ± 0.049) (Figure 4D). These results indicated that LIPUS‐SCs‐Exo could improve erectile function.

### 3.7 Histological changes in corpus cavernosum

After measuring the erectile function of the rats, H&E and Masson staining examined the histological changes of the rat corpus cavernosum (Figure 4E,G). We assessed the degree of corpus cavernous sinus atrophy and fibrosis by the ratios of lumen to parenchyma (Figure 4F) and smooth muscle to collagen, respectively (Figure 4H). The BCNI group had a significantly lower ratios of lumen to parenchyma and smooth muscle to collagen compared to the control group. The ratios of the SCs‐Exo group and LIPUS‐SCs‐Exo group was increased compared to the BCNI group, and the latter ratios was higher.

### 3.8 LIPUS‐SCs‐Exo promotes smooth muscle cell and endothelial cell function recovery

**FIGURE 5:**
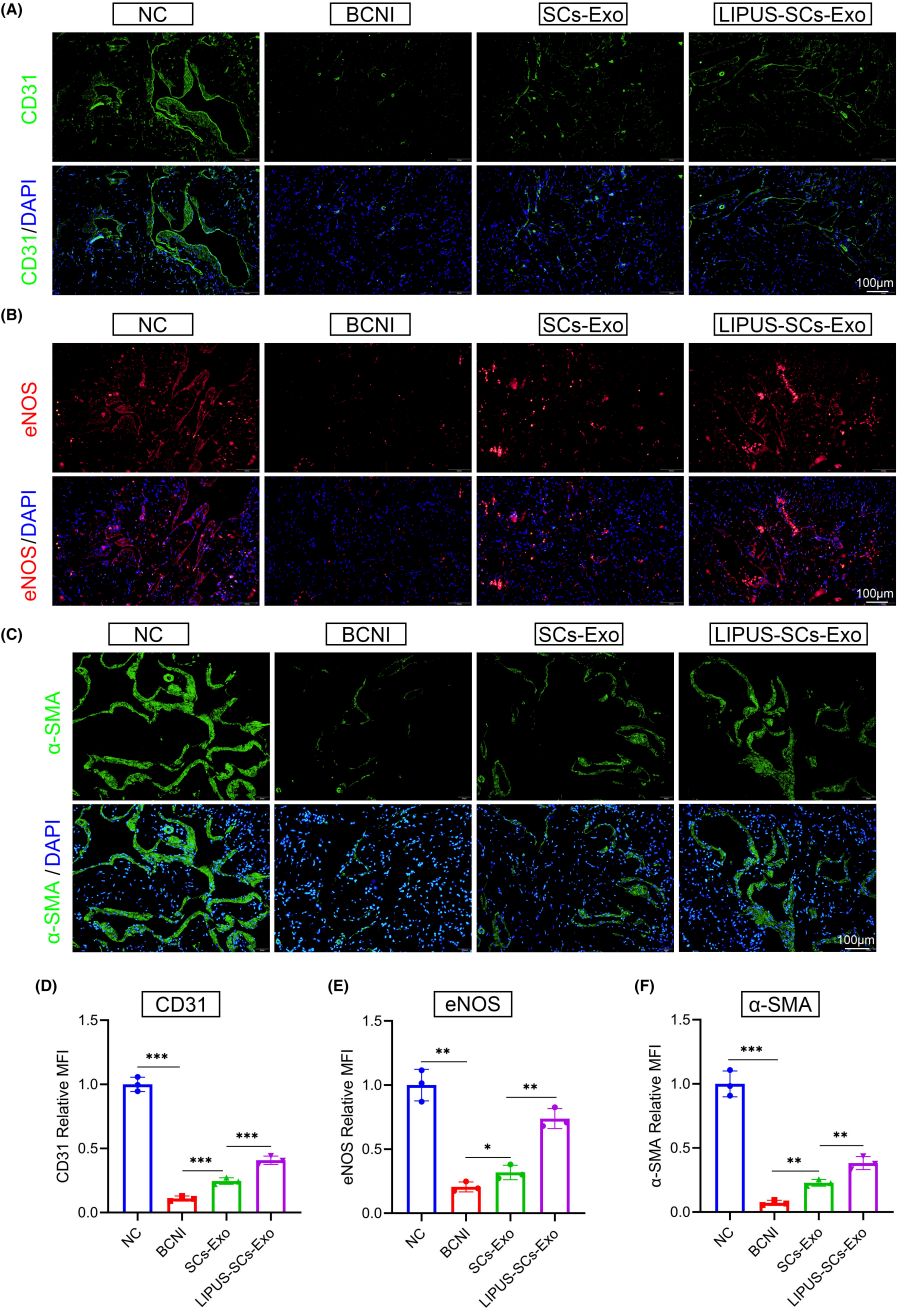
The effect of LIPUS‐SCs‐Exo on endothelial and smooth muscle cell contents and function. Endothelium cell content and function in corpus cavernosum were evaluated by immunofluorescent staining for CD31 (green) (A) and eNOS (red) (B), respectively. The nucleus was stained with DAPI (blue). (Scale bar = 100 μm.) Semi‐quantitative image analysis of the relative MFI of CD31 (D) and eNOS (E). Data presented as mean ± SD with three replicates. *p < 0.05; **p < 0.01; ***p < 0.001. (C) Smooth muscle cell content in corpus cavernosum were evaluated by immunofluorescent staining for α‐SMA (green), DAPI (blue) and Merge file. (Scale bar = 100 μm.) (F) Semi‐quantitative image analysis of the relative MFI of α‐SMA. Data presented as mean ± SD with three replicates. **p < 0.01; ***p < 0.001. Exo, exosomes; LIPUS, low‐intensity pulsed ultrasound; MFI, mean fluorescence intensity; SCs, Schwann cells.

To evaluate the effect of LIPUS‐SCs‐Exo on endothelial and smooth muscle cell contents in the corpus cavernosum, we used immunofluorescence to detect the expression change of their marker genes, respectively (Figure 5A–C). The BCNI group had significantly lower levels of the smooth muscle marker α‐SMA levels (Figure 5C) and the endothelial cell markers CD31 and eNOS levels (Figure 5A,B) in the BCNI group compared to the normal control group. The α‐SMA, CD31, and eNOS levels in the SCs‐Exo group and LIPUS‐SCs‐Exo group recovered significantly, with a greater recovery in the LIPUS‐SCs‐Exo group than in the SCs‐Exo group (Figure 5D–F).

### 3.9 LIPUS‐SCs‐Exo promotes nerve regeneration in vivo

**FIGURE 6:**
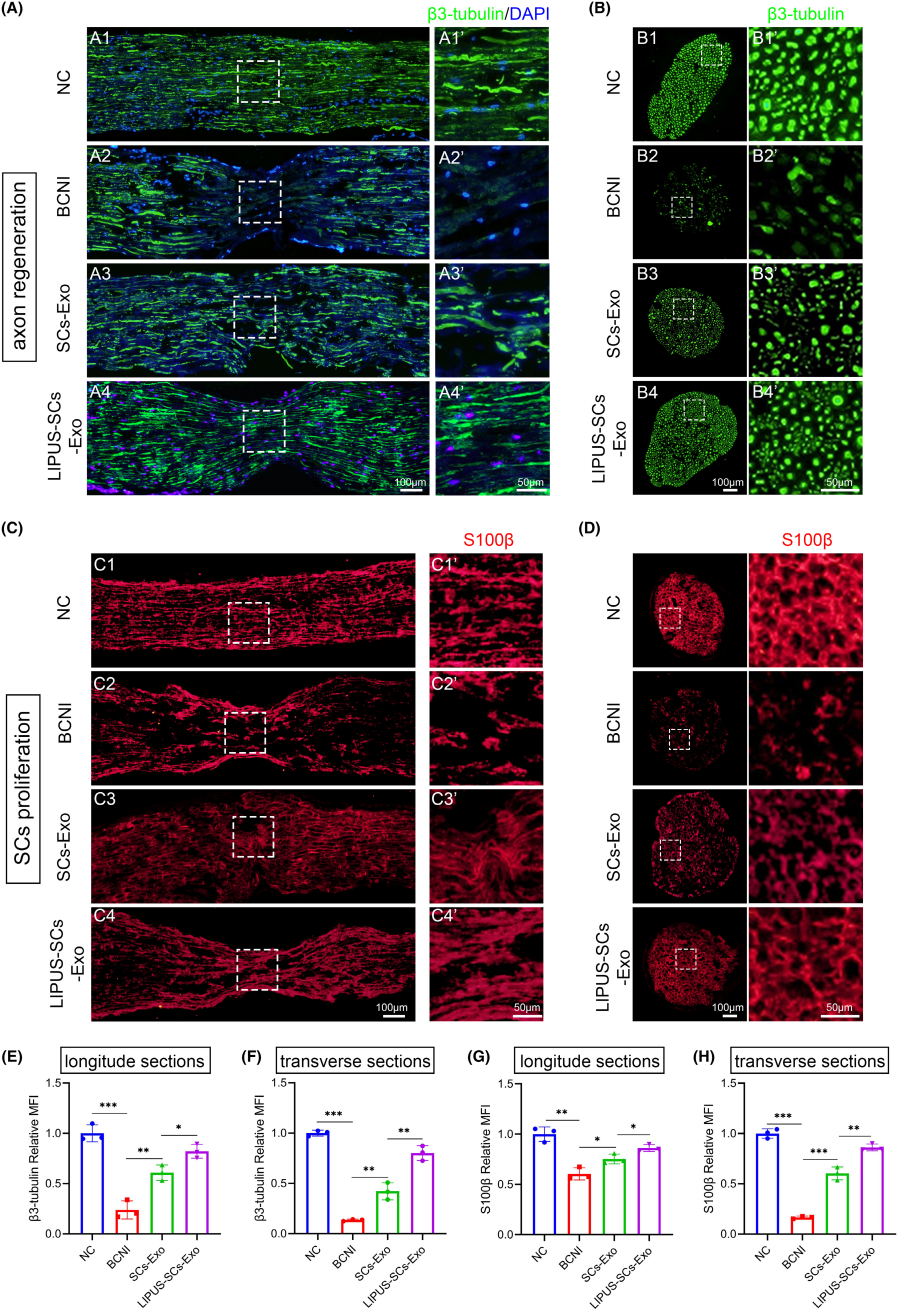
The effect of LIPUS‐SCs‐Exo on axon regeneration and SCs proliferation in vivo. Representative immunostaining images of CN longitude sections were stained for β3‐tubulin (green) (A) and S100β (red) (C) in each group, respectively. The nucleus was stained with DAPI (blue). (Scale bar = 100 μm.) Representative immunostaining images of CN transverse sections of the distal regenerated nerve segments were stained for β3‐tubulin (green) (B) and S100β (red) (D) in each group, respectively. The nucleus was stained with DAPI (blue). (Scale bar = 100 μm.) Semi‐quantitative image analysis of the β3‐tubulin relative MFI of CN longitude sections (E) and transverse sections (F). Data presented as mean ± SD with three replicates. *p < 0.05; **p < 0.01; ***p < 0.001. Semi‐quantitative image analysis of the S100β relative MFI of CN longitude sections (G) and transverse sections (H). Data presented as mean ± SD with three replicates. *p < 0.05; **p < 0.01; ***p < 0.001. Exo: exosomes; LIPUS, low‐intensity pulsed ultrasound; MFI, mean fluorescence intensity; SCs, Schwann cells.

After identifying that LIPUS‐SCs‐Exo promotes axonal growth in vitro, we examined their effects on axon regeneration and SCs proliferation after CN injury in vivo. We stained the CN with immunofluorescence using the neuronal marker β3‐tubulin (Figure 6A) and the SCs marker S100β (Figure 6C). We observed that the LIPUS‐SCs‐Exo group had stronger axonal regeneration ability than the BCNI and SCs‐Exo groups (Figure 6E). Moreover, the S100β level in the LIPUS‐SCs‐Exo group was increased at the injured nerve stage, indicating that SCs proliferation was stronger (Figure 6G). Additionally, the transverse‐section of the distal regenerated nerve segments was analyzed using immunofluorescence staining (Figure 6B,D). The S100β and β3‐tubulin expression levels in the LIPUS‐SCs‐Exo group were significantly increased compared to SCs‐Exo and BCNI groups (Figure 6F,H). These results indicated that LIPUS‐SCs‐Exo might stimulate SCs proliferation, which was more conducive to axon regeneration.

### 3.10 The miRNA expression in SCs‐Exo and LIPUS‐SCs‐Exo

**FIGURE 7:**
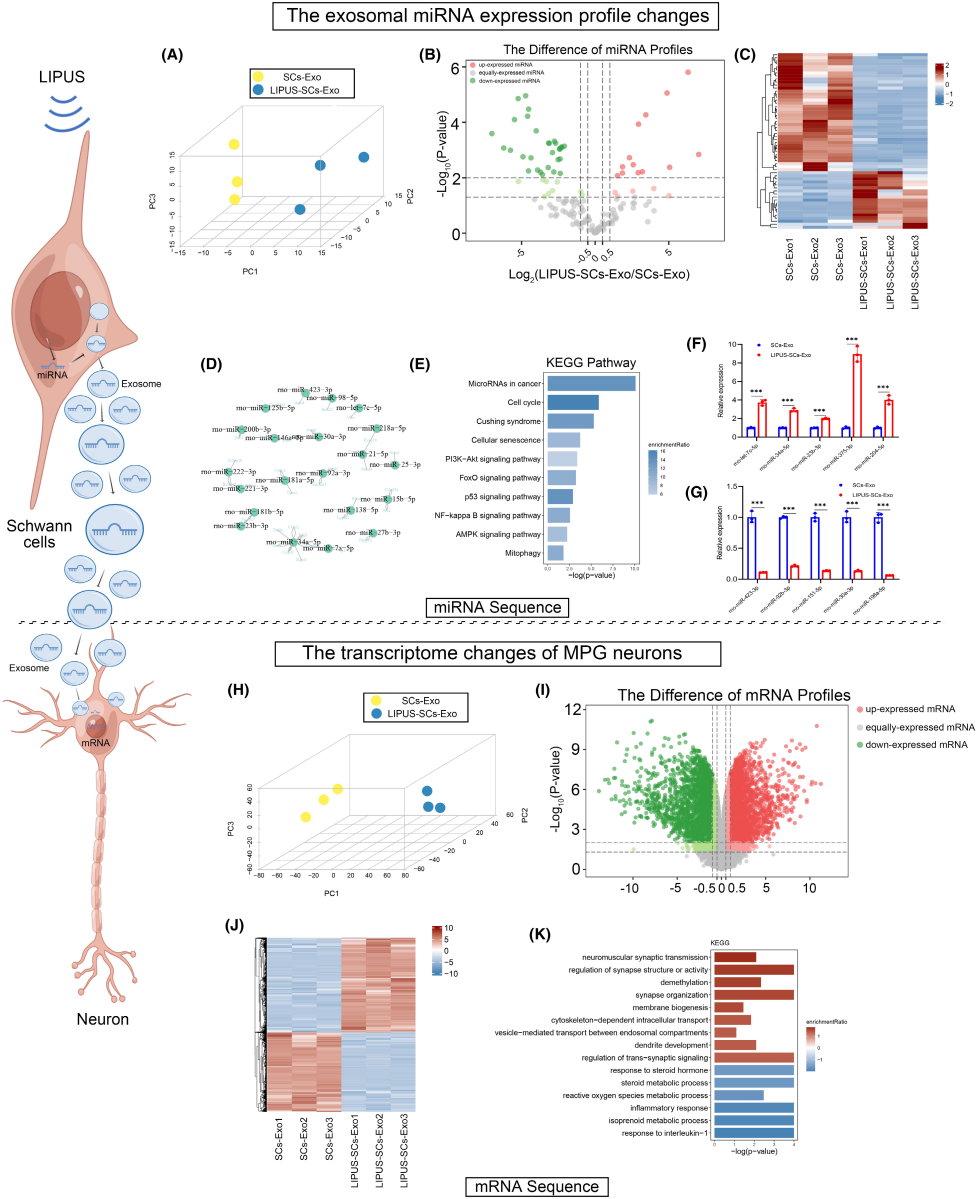
The exosomal miRNA expression profile changes and the transcriptome changes of MPG neurons after SCs‐Exo and LIPUS‐SCs‐Exo treatment. (A) PCA 3D mapping plot of miRNA expression profile in each group. (B) Volcano plot showed the differentially expressed miRNAs between LIPUS‐SCs‐Exo and SCs‐Exo. A gradient of green to red indicated the down‐expressed to up‐regulation of the miRNAs. (C) Heatmap diagram showed the expression level of differentially expressed miRNAs between LIPUS‐SCs‐Exo and SCs‐Exo. A gradient of blue to red indicated the down‐expressed to up‐regulation of the miRNAs. (D) Target gene network of differentially expressed miRNAs between LIPUS‐SCs‐Exo and SCs‐Exo. (E) KEGG pathway enrichment of target genes of the predicted differentially expressed miRNAs. Confirmation of the up‐expressed miRNA (F) and down‐expressed miRNAs (G) in LIPUS‐SCs‐Exo compared to SCs‐Exo by qRT‐PCR. The data were presented as mean ± SD. *n* = 3; ***p < 0.001. (H) PCA 3D mapping plot of DEGs profile in each group. (I) Volcano plot showed the DEGs in each group. A gradient of green to red indicated the down‐expressed to up‐regulation of the genes. (J) Heatmap diagram showed the expression level of DEGs in each group. A gradient of blue to red indicated the down‐expressed to up‐regulation of the genes. (K) KEGG pathway analysis of DEGs. DEGs, differentially expressed genes; Exo, exosomes; KEGG, Kyoto Encyclopedia of Genes and Genomes; LIPUS, low‐intensity pulsed ultrasound; PCA, principal component analysis; SCs, Schwann cells.

Exosome‐enriched microRNAs are essential for intercellular communication in the nervous system.^25^, ^26^, ^29^ We conducted high‐throughput sequencing on miRNA expression profiles of LIPUS‐SCs‐Exo and SCs‐Exo to decode the molecular mechanism of LIPUS‐SCs‐Exo promoting nerve regeneration. We evaluated the reproducibility of the samples (Figure 7A) and compared the miRNA expression levels between LIPUS‐SCs‐Exo and SCs‐Exo (Figure 7B,C). We employed qRT‐PCR to detect the changes of 10 miRNAs between LIPUS‐SCs‐Exo and SCs‐Exo with log_2_ (Fold change) absolute value ≥3 and *p* < 0.05 as cut‐off thresholds (Table 2). The results indicated that the sequencing was accurate (Figure 7F,G). We utilized a computational target prediction algorithm, miRWalk, to predict target genes of differential miRNAs (Figure 7D). The KEGG pathway was performed to evaluate its potential biological characteristics (Figure 7E). We discovered that pathway enrichment includes PI3K‐Akt, AMPK, and FoxO signaling pathways.

**TABLE 2:** The top five expression level alters miRNAs in LIPUS‐SCs‐Exo compared to SCs‐Exo.

miRNA	log_2_(fold change)	*p* value	LIPUS‐SCs‐Exo Expression	SCs‐Exo Expression
Up‐regulated				
rno‐let‐7c‐5p	5.4681	1.56E‐49	136.332	4.67
rno‐miR‐34a‐5p	4.0530	3.84E‐27	23.656	2.192
rno‐miR‐23b‐3p	3.3182	1.95E‐07	8.367	1.295
rno‐miR‐375‐3p	7.1164	5.18E‐05	9.51	0.082
rno‐miR‐204‐5p	5.5635	9.71E‐07	3.673	0.11
Down‐regulated				
rno‐miR‐423–3p	−4.6208	3.74E‐38	5.601	210.062
rno‐miR‐92b‐3p	−3.8813	8.47E‐19	1.366	30.933
rno‐miR‐151‐5p	−4.0823	2.57E‐20	1.094	28.476
rno‐miR‐30a‐3p	−3.9239	9.12E‐04	0.454	11.216
rno‐miR‐196a‐5p	−6.6641	4.00E‐12	0.065	10.172

### 3.11 The mRNA expression profiles from MPG neurons treated by SCs‐Exo and LIPUS‐SCs‐Exo

To explore further the molecular mechanism of LIPUS‐SCs‐Exo promoting nerve regeneration, we conducted high‐throughput sequencing on the mRNA expression profiles of MPG neurons treated with LIPUS‐SCs‐Exo or SCs‐Exo. Similarly, we evaluated the reproducibility of the samples (Figure 7H) and compared the mRNA expression levels between LIPUS‐SCs‐Exo group and SCs‐Exo group (Figure 7I,J). The KEGG pathway was used to analyze the enriched signals, which were related to synaptic structure and vesicle transport regulation (Figure 7K).

### 3.12 LIPUS‐SCs‐Exo promotes nerve regeneration via PI3K/Akt/FoxO signaling pathway

**FIGURE 8:**
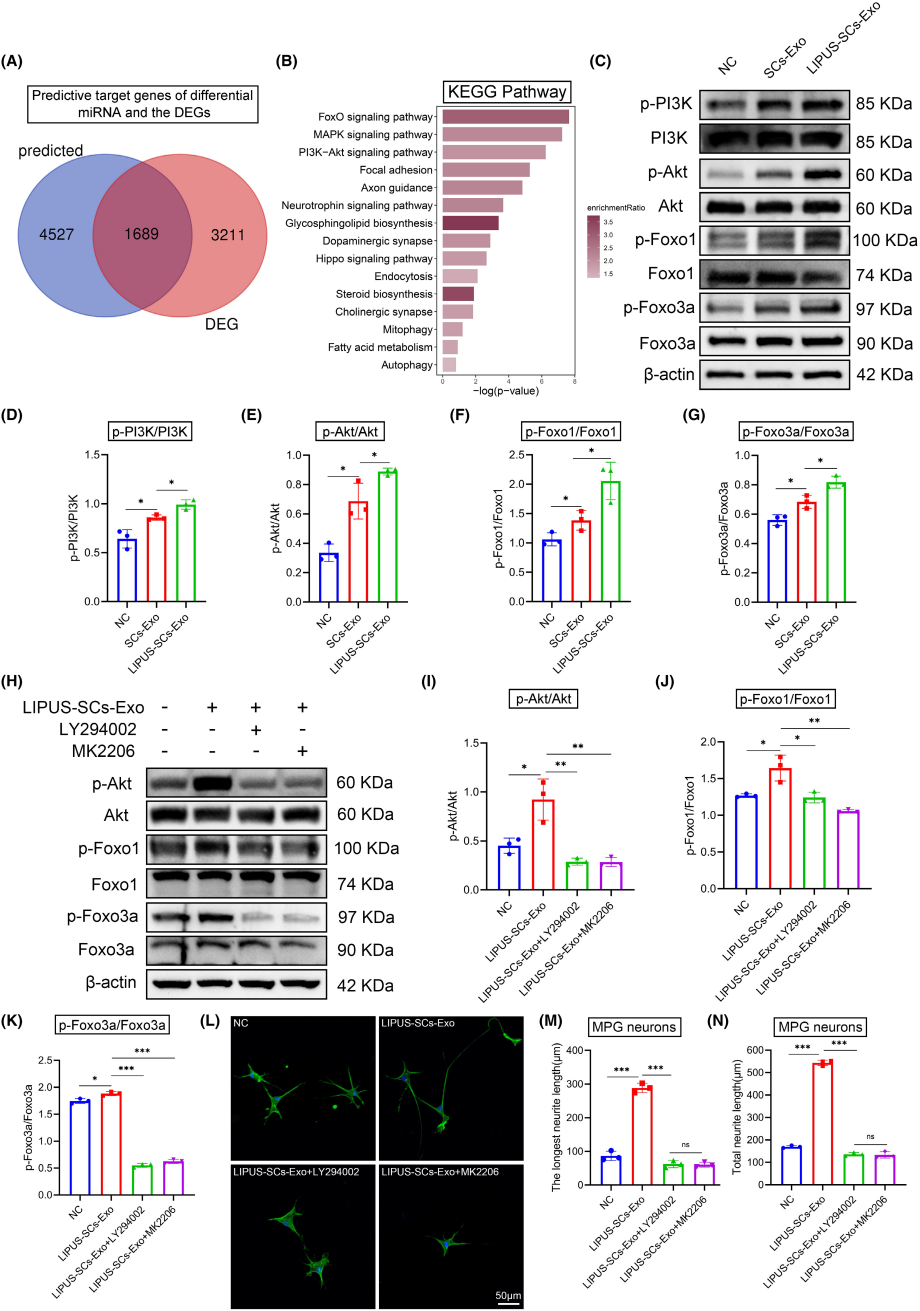
LIPUS‐SCs‐Exo promotes nerve regeneration via PI3K/Akt/FoxO signaling pathway. (A)Venn diagram showed 1689 key genes between predictive target genes of differential miRNA and the DEGs after LIPUS‐SCs‐Exo and SCs‐Exo treatment. (B) KEGG pathway enrichment of the key genes. (C) MPG neurons were treated with SCs‐Exo or LIPUS‐SCs‐Exo for 72 h. Western Blotting was used to detect the expression of PI3K, p‐PI3K, Akt, p‐Akt, FoxO1, p‐FoxO1, FoxO3a and p‐FoxO3a in MPG neurons. The quantization of protein expression levels. The ratios of p‐PI3K/PI3K (D), p‐Akt/Akt (E), p‐FoxO1/FoxO1 (F), p‐FoxO3a/FoxO3a (G) of every group were calculated with β‐actin as an internal reference. Data presented as mean ± SD with three replicates. *p < 0.05. MPG neurons were pretreated with LY294002 (10 μM) and MK2206 (1 μM) for 3 h, and then treated with LIPUS‐SCs‐Exo for 72 h. (H) The activation of PI3K‐Akt was examined with Western Blotting. (I–K) The quantization of PI3K‐Akt‐FoxO protein expression levels. Data presented as mean ± SD with three replicates. *p < 0.05; **p < 0.01; ***p < 0.001. (L) Inhibition of PI3K‐Akt signal in MPG neurons partially counteracted the enhancement of growth by LIPUS‐SCs‐Exo. Quantification of neurites is shown in panel (M) and panel (N). Data presented as mean ± SD with three replicates. Ns, no significant difference; *n* = 3; ***p < 0.001. DEGs, differentially expressed genes; Exo, exosomes; KEGG, Kyoto Encyclopedia of Genes and Genomes; LIPUS, low‐intensity pulsed ultrasound; MPG, major pelvic ganglion; SCs, Schwann cells.

We screened 1689 predictive target genes of differential miRNA in LIPUS‐SCs‐Exo and SCs‐Exo groups and the neuron differential genes after treatment in the two groups based on the results of two high‐throughput sequencings (Figure 8A). Subsequently, we conducted a KEGG analysis on these 1689 genes. The enriched signal pathway was associated with axon regeneration, including FoxO, MAPK, PI3K‐Akt, axon guidance, and neurotrophin signaling pathways (Figure 8B). FoxO transcription factor is an important downstream molecule of the PI3K‐AKT signal pathway and receives negative regulation.^30^ We measured the activation of the PI3K‐Akt‐FoxO signaling pathway in MPG neurons to elucidate the molecular mechanism of LIPUS‐SCs‐Exo promoting axonal regeneration (Figure 8C). Figure 8D,E illustrate that LIPUS‐SCs‐Exo significantly increased the phosphorylated levels of PI3K and Akt compared to NC and SCs‐Exo groups. In addition, Akt can also directly phosphorylate the transcription factor FoxO.^30^ The phosphorylated FoxO1 and FoxO3a in the LIPUS‐SCs‐Exo group were remarkably increased compared to those in the NC and SCs‐Exo groups (Figure 8F,G).

Furthermore, we used a specific PI3K inhibitor (LY294002, 10 μM) and a specific Akt inhibitor (MK2206, 1 μM), preconditioning MPG neurons for 3 h, and then treated with LIPUS‐SCs‐Exo (Figure 8H). Both inhibitors can significantly reduce Akt and FoxO phosphorylation (Figure 8I–K). Moreover, the inhibitor counteracted the function of LIPUS‐SCs‐Exo to promote MPG neuron axon elongation (Figure 8L–N). These results indicated that the ability of LIPUS‐SCs‐Exo to promote axon regeneration was associated with the PI3K‐Akt‐FoxO signaling pathway, and inhibition of PI3K‐Akt‐FoxO pathway activity could significantly inhibit the role of LIPUS‐SCs‐Exo in promoting axon regeneration.

## 4 DISCUSSION

The NED incidence is 14–90% due to CN injury in pelvic surgery, such as RP.^31^ Therefore, finding a safe and effective method to promote the regeneration of damaged CN is the difficult hotspot of current research. Our study discovered that LIPUS‐SCs‐Exo promotes CN regeneration and restores erectile function in BCNI rats. We performed exosomal miRNA sequencing and target cell neuronal mRNA sequencing, combined with bioinformatics analysis and experimental validation, to further investigate the molecular mechanism and finally identified the key role of the PI3K‐Akt‐FoxO signaling pathway in nerve regeneration.

Many studies have demonstrated that LIPUS, as a non‐invasive micro‐energy biological treatment measure, promotes SCs proliferation and peripheral nerve regeneration.^19^, ^26^, ^32^ This study established an in vitro MPG/CN explant culture model to simulate axonal growth after CN injury. We demonstrated that LIPUS could promote the axon elongation of CN breakpoints in an energy‐dependent manner using this model. SCs are the key cells for regeneration after PNI. They can be reprogrammed to repair phenotype and guide axon regeneration.^17^ According to recent studies, SCs are important target cells for sensing LIPUS stimulation.^14^, ^32^ Therefore, LIPUS ability to promote axonal regeneration of MPG/CN explants may be related to the activation of SCs.

CN differs structurally and functionally from other peripheral nerves, with a unique distribution of sympathetic and parasympathetic fibers.^33^ CN is located on the dorsolateral aspect of the prostate. Its primary function is to promote vasodilation of the corpus cavernosum and cooperate with the perineal nerve to erect the penis.^34^ The CN nerve fibers are not completely myelinated, and some of them are wrapped into Remak bundles by non‐myelinated SCs.^35^ Therefore, considering the possible different biological functions of cavernous neural SCs, we extracted primary CN‐derived SCs from SD rats for subsequent studies. Previous studies have demonstrated that micro‐energy mechanical stimulation changes the phenotype of SCs and promotes their proliferation and migration capabilities.^27^, ^32^, ^36^ This study evaluated whether LIPUS had a similar effect on CN‐derived SCs. We discovered that LIPUS at low energy intensities promoted CN‐derived SCs viability and proliferation using MTT and EdU assays. Moreover, MPG neurons co‐incubated with SCs pretreated with LIPUS significantly promoted axon elongation, indicating that SCs are the key cells receiving mechanical stimulation, and the activated SCs are likely to change the paracrine cargos.

Intercellular communication is essential in maintaining physiological homeostasis, and exosomes play a crucial role in this process. Exosomes derived from SCs play a key role in regulating nerve regeneration in the nervous system.^29^ Exosomes derived from SCs may promote SCs dedifferentiation and proliferation after nerve injury.^37^ Recent evidence demonstrated that axons could internalize exosomes and enhance neurite outgrowth.^38^ These studies are similar to our results. We revealed that SCs release LIPUS‐SCs‐Exo, and MPG neurons internalize it.

Moreover, neuronal cytoplasm and neurite contained internalized exosomes. This study demonstrated that SCs‐Exo promoted nerve regeneration and improved erectile function in BCNI rats. Moreover, LIPUS‐SCs‐Exo had a stronger ability to promote axonal elongation and restore erectile function. This finding indicated that LIPUS‐SCs‐Exo might have important therapeutic value. When LIPUS stimulates SCs, the exosome cargos released by the SCs change. Therefore, clarifying its potential mechanism may provide a new direction for CN axon regeneration and NED treatment.

Recent studies focus on exosomal miRNA function in the nervous system.^25^, ^39^, ^40^ In the central nervous system, exosomal miR‐199a‐3p/145‐5p derived from human umbilical cord mesenchymal stem cells promotes locomotor function recovery in rats with spinal cord injury by up‐regulating the TrkA expression.^39^ Furthermore, fibroblast‐derived exosomal miR‐673‐5p can promote myelin gene expression in SCs, which is important for peripheral myelination in the PNS.^25^ The miRNA expression profiles of adipose‐derived mesenchymal stem cell exosomes differ depending on differentiation level. Among them, miR‐132‐3p and miR‐199b‐5p are highly associated with neuroprotection and angiogenesis.^41^

We conducted high‐throughput miRNA sequencing to determine the differential miRNA expression profile of LIPUS‐SCs‐Exo and SCs‐Exo. A total of 68 differential miRNAs were obtained, among which several miRNAs have been reported to play important roles in various biological processes, such as axonal growth and neurological diseases.^26^, ^42^, ^43^ For example, Xia et al. found that mechanical stimuli can upregulate the expression of miR‐23b‐3p in SCs derived extracellular vesicles, decreasing neuronal Nrp1 expression and promoting axonal regeneration.^26^ Through bioinformatics analysis, Su et al. found that miR‐204‐5p and several transcription factors (such as Smada2, Fli1, Wt1, Sp6, and Sp3) play an important role in axonal regeneration.^42^ Tan et al. reported that loss of miR‐30a‐3p enhanced EP300 and BDNF colocalization in sciatic nerve chronic constrictive injury rats and is associated with the progression of neuropathic pain.^43^ Therefore, we considered that the axonal regeneration of MPG neurons was more likely to be affected by the combined effects of several miRNAs in LIPUS‐SCs‐Exo. Limitation to one or several miRNAs and target mRNA as regulatory signals did not reflect the real situation in vivo. Through pathway enrichment of the above 1689 genes and comprehensive analysis of their biological effects, we speculated that the miRNA of LIPUS‐SCs‐Exo may play a role in promoting axonal regeneration by upregulating the PI3K‐Akt‐FoxO signaling pathway in MPG neurons. Subsequently, our results indicated that the PI3K‐Akt‐FoxO signaling pathway played a crucial role in axon regeneration because inhibition of PI3K‐Akt‐FoxO pathway almost eliminated the function of LIPUS‐SCs‐Exo to promote neurite outgrowth.

PI3K‐Akt signaling pathway regulates numerous cellular physiological functions, such as proliferation and growth.^44^, ^45^ The PI3K‐Akt pathway inhibits cell proliferation by negatively regulating the FoxO transcription factor.^30^ FoxO protein specifically binds to 14‐3‐3 protein, resulting in conformational changes after being phosphorylated by Akt, thereby masking nuclear localization signals and exposing nuclear export signals.^46^ Several studies reported that the prosurvival/proangiogenic PI3k‐Akt‐FoxO signaling mediated the therapeutic benefits of nerve growth factor transfer.^47^, ^48^ Our results revealed that LIPUS‐SCs‐Exo treatment could increase the phosphorylated level of PI3k, Akt, FoxO1, and FoxO3a. When PI3K and Akt‐specific inhibitors were used, the phosphorylated level of FoxO1 and FoxO3a would decrease.

Moreover, PI3K and Akt‐specific inhibitors may counteract LIPUS‐SCs‐Exo function in promoting neurite outgrowth. Therefore, we considered that PI3K was activated after MPG neurons were treated with LIPUS‐SCs‐Exo. Then, Akt was phosphorylated and transferred to the nucleus to regulate FoxO transcription factors. The main mechanism involved FoxO subcellular localization and phosphorylation. These findings suggested that LIPUS‐SCs‐Exo‐derived miRNAs can effectively regulate axon regeneration, and the PI3K‐Akt‐FoxO pathway is a crucial signal.

In summary, this study extracted primary SCs from CN for the first time and demonstrated that LIPUS stimulation significantly affects the miRNA composition in SCs‐derived exosomes. According to high‐throughput sequencing and bioinformatics analysis, LIPUS‐SCs‐Exo can promote nerve regeneration by activating the PI3K‐Akt‐FoxO signaling pathway. Our study has several limitations; our exosomal cargos focused primarily on miRNA alterations; in the future, we might investigate DNA, mRNA, lncRNA, circRNA, and protein. This study has the potential to provide a non‐invasive and effective treatment for ED caused by CN injury. These findings revealed that SCs could sense LIPUS mechanical signals and transduce mechanical signals via intercellular communication between exosomes and neurons. However, how LIPUS converts mechanical signals into biochemical signals to SCs remains unclear. This mechanobiological signaling may be related to the integrin signaling pathway,^49^ a mechanosensitive ion channel,^50^ which will be one of the focuses of our next plan. Exosomes have been demonstrated to be a novel nanotherapeutic carrier. Recently, the application of engineered exosomes for diagnosis and treatment has become a research hotspot.^51^, ^52^ According to our current study, how to engineer LIPUS‐SCs‐Exo for clinical translation is a hot and difficult point in the future.

## AUTHOR CONTRIBUTIONS

Kun Ye, Liangyu Zhao and Yuxin Tang conceived this study; Kun Ye, Yinghao Yin and Dongyi Peng designed the methods; Kun Ye and Zitaiyu Li performed the majority of experiments and analyzed the data; Jun Zhou and Ming Xiao collected the data and performed the statistical analysis; Dongjie Li and Yu Gan designed the experimental procedures; Kun Ye wrote the original manuscript; Yuxin Tang, Liangyu Zhao and Yingbo dai reviewed and edited the manuscript. All authors have read and approved the final version of the manuscript.

## FUNDING INFORMATION

This work was supported by the National Natural Science Foundations of China (82071636, Y.T.), Guangdong Basic and Applied Basic Research Foundation (2021A1515010436, Y.T.; and 2021A1515111109, L.Z.), China Postdoctoral Science Foundation (2021M703747, L.Z.), and Open Funds of Guangdong Provincial Key Lab of Biomedical Imaging (GPKLBI202104, L.Z.).

## CONFLICT OF INTEREST STATEMENT

The authors declare no conflict of interest.

## Supporting information


Data S1

## Data Availability

All miRNA‐seq and mRNA‐seq raw data generated for this study have been submitted in the Sequence Read Article (SRA, https://www.ncbi.nlm.nih.gov/sra/) “SRR24032731, SRR24032732, SRR24032733, SRR24032734, SRR24032735, SRR24032736, SRR24037503, SRR24037504, SRR24037505, SRR24037506, SRR24037507, SRR24037508” on April 1, 2023. All other relevant data supporting the key findings of this study are available within the article and its Supplementary Information files or from the corresponding author upon reasonable request.
