# Supplementary figures and images for: LIPUS‐SCs‐Exo promotes peripheral nerve regeneration in cavernous nerve crush injury‐induced ED rats via PI3K/Akt/FoxO signaling pathway
Source: CNS Neurosci Ther. 2023 May 8;29(11):3239–58. doi: 10.1111/cns.14256 (PMC10580359; doi:10.1111/cns.14256)

## Full unedited blot for Figure 3C

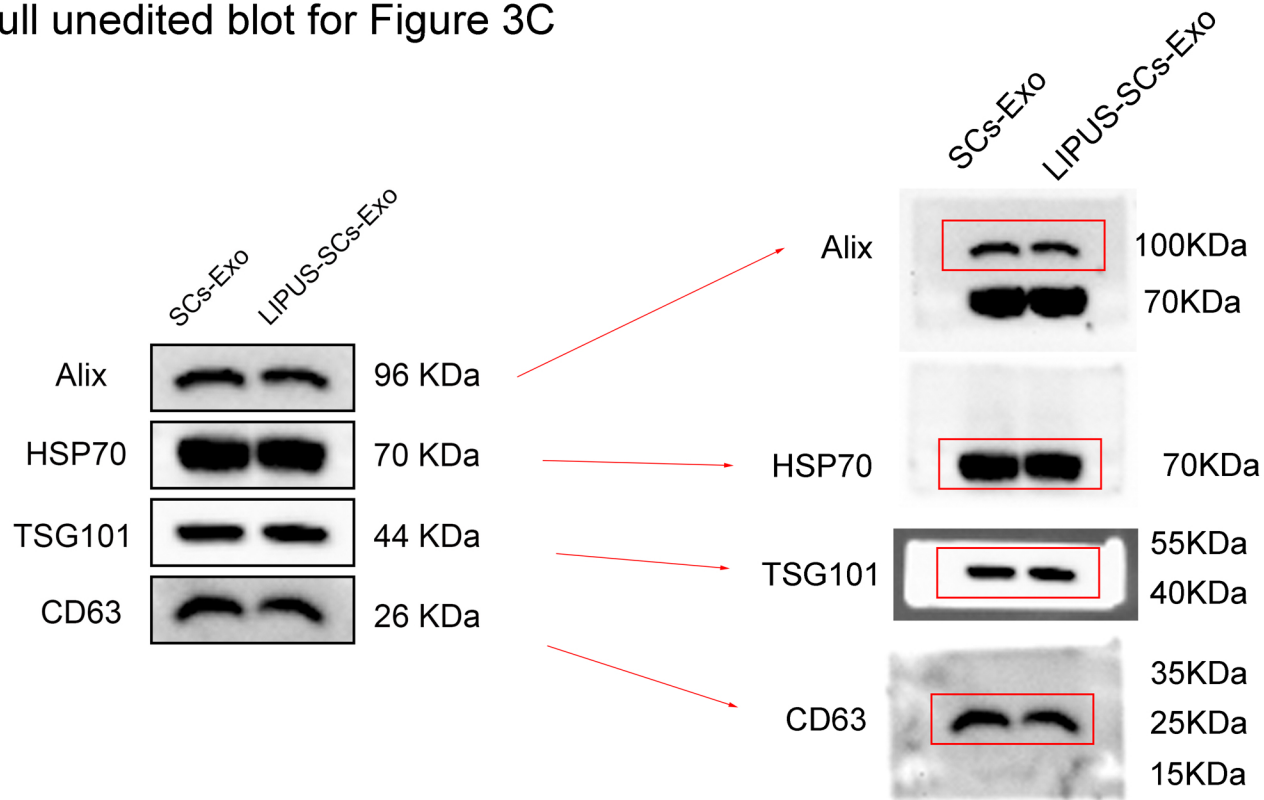

Full unedited blot for Figure 8C

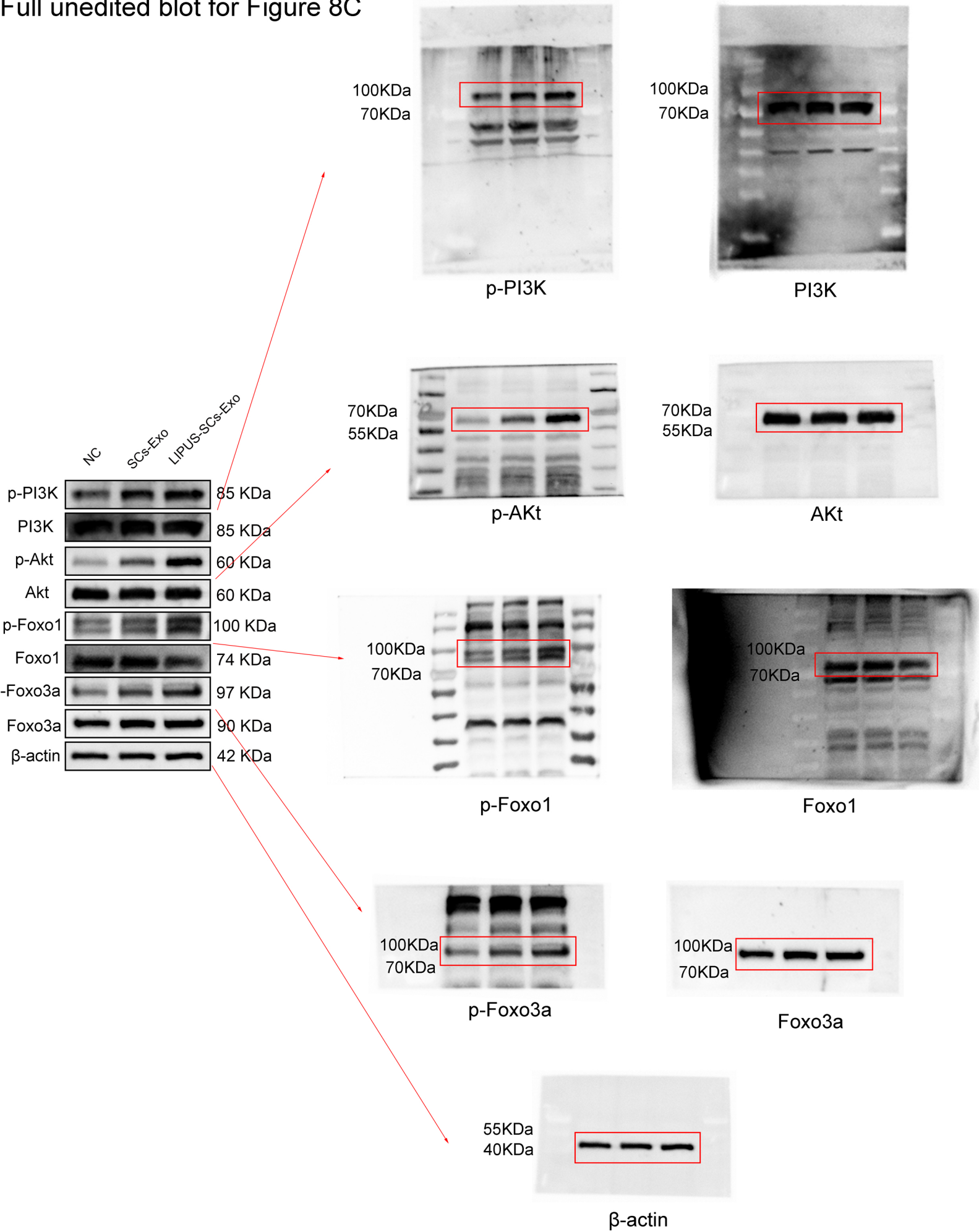

Full unedited blot for Figure 8H

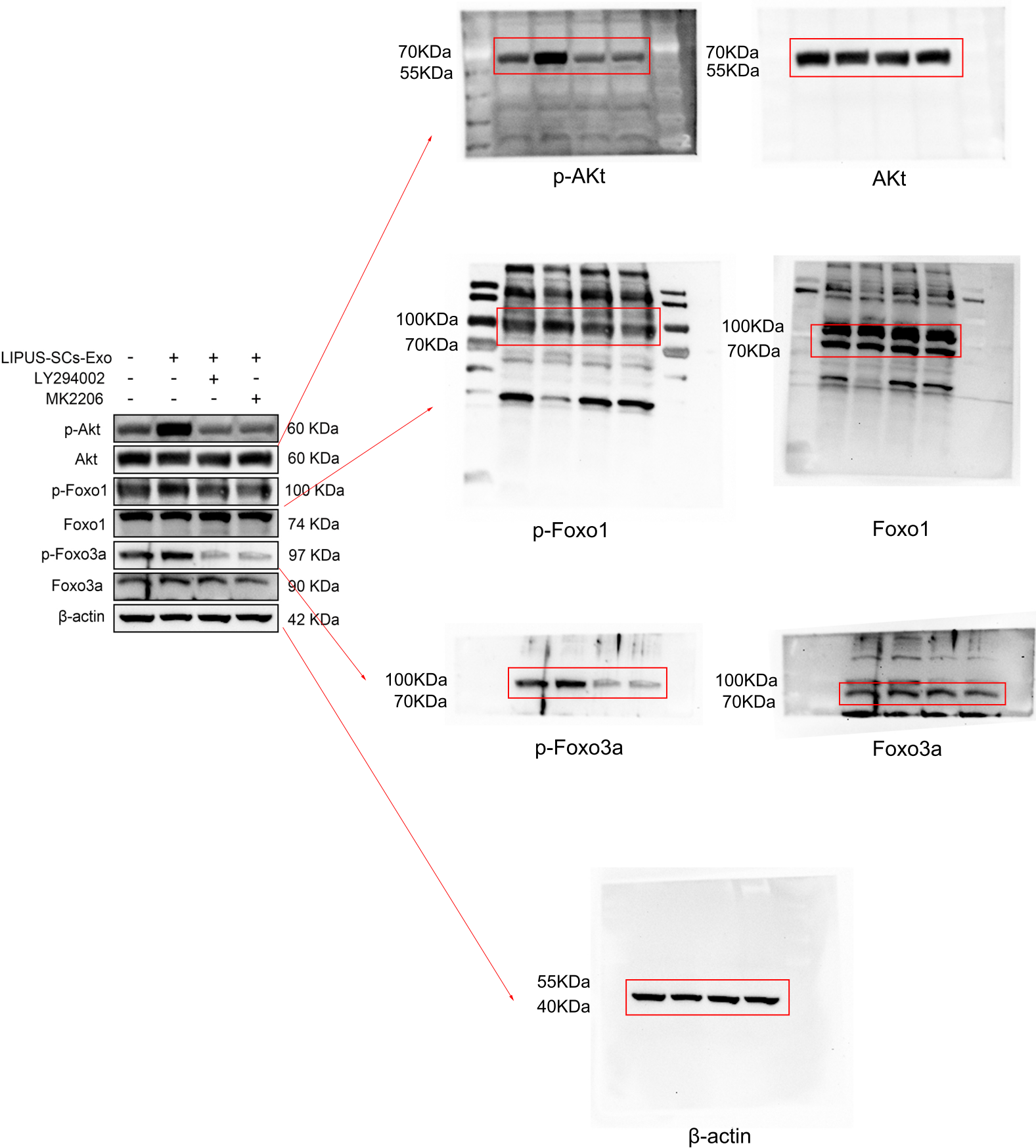

Supplement: Supplementary file 1 — Data S1 [file CNS-29-3239-s001.pdf]
